# Formal synthesis of dibenzotetrathiafulvalene (DBTTF), through practical electrochemical preparation of benzo[d]-1,3-dithiole-2-one (BDTO)

**DOI:** 10.3389/fchem.2025.1666772

**Published:** 2025-09-04

**Authors:** Álvaro V. Terán-Alcocer, Fernanda M. J. Cifuentes-Ajuchan, Byron J. López-Mayorga, Bernardo A. Frontana-Uribe

**Affiliations:** 1 Centro Conjunto de Investigación en Química Sustentable UAEMex-UNAM, Carretera Toluca-Ixtlahuaca Km 14.5, Toluca, Estado de México, Mexico; 2 Instituto de Química, Universidad Nacional Autónoma de México Circuito Exterior, Ciudad Universitaria, Coyoacán, Mexico; 3 Facultad de Ciencias Químicas y Farmacia, Universidad de San Carlos de Guatemala, Ciudad Universitaria, Caracas, Guatemala

**Keywords:** DBTTF, benzo[d]-1,3-dithiole-2-ones (BDTO), electrosynthesis, electrochemical cyclization, O-ethyl-S-phenyldithiocarbonate

## Abstract

This study focuses on developing an electrochemical reaction to produce benzo[d]-1,3-dithiole-2-one (BDTO). This compound serves as a direct precursor for dibenzotetrathiafulvalenes (DBTTF), an important member of the charge transfer complex family. BDTO is synthesized in three steps (16%) starting from aniline. The key reaction is an anodically driven intramolecular cyclization (35%), involving a thiyl radical-cation intermediate formed from the oxidation of the S-aryl-O-ethyldithiocarbonate derivative. This derivative is obtained in good yields from its respective aromatic diazonium salt. This approach eliminates the need for advanced, costly intermediates and avoids long, complex synthetic routes previously used to produce BDTO, utilizing safer and cheaper reagents. This opens the door to generating DBTTF derivatives quickly.

## 1 Introduction

The tetrathiafulvalene (TTF) molecule and its derivatives have attracted the attention of scientists since the early 1970s due to their attractive properties as a conductive material (Andrieux et al., 1979; Melby et al., 1974; Yoneda et al., 1979). When it forms a charge transfer complex with the tetracyanoquinodimethane molecule (TTF-TCNQ) an organic metal is obtained since its conductivity reaches 10^4^ S cm^-1^ at 59 K (Ferraris et al., 1973; Metz, 1973). The dibenzotetrathiafulvalene (DBTTF) family represents a great alternative to TTFs due to its ability to modulate redox potential by introducing functional groups in the benzyl ring. Their diverse applications are innumerable, and they play an important role as redox sites in different areas such as sensors, Balandier et al. (2007) starting materials for organic electronic systems, Gao et al. (2006) molecular redox switches, as a building block in supramolecular architectures, Jana et al. (2017) and many others. However, further development of these materials has been limited by a complicated or expensive synthesis route to prepare the final intermediaries of synthesis, such as benzo[d]-1,3-dithiole-2-ones (BDTO) (**1**) and benzo[d]-1,3-dithiole-2-thiones (BDTT) (**2**).

**SCHEME 1:**
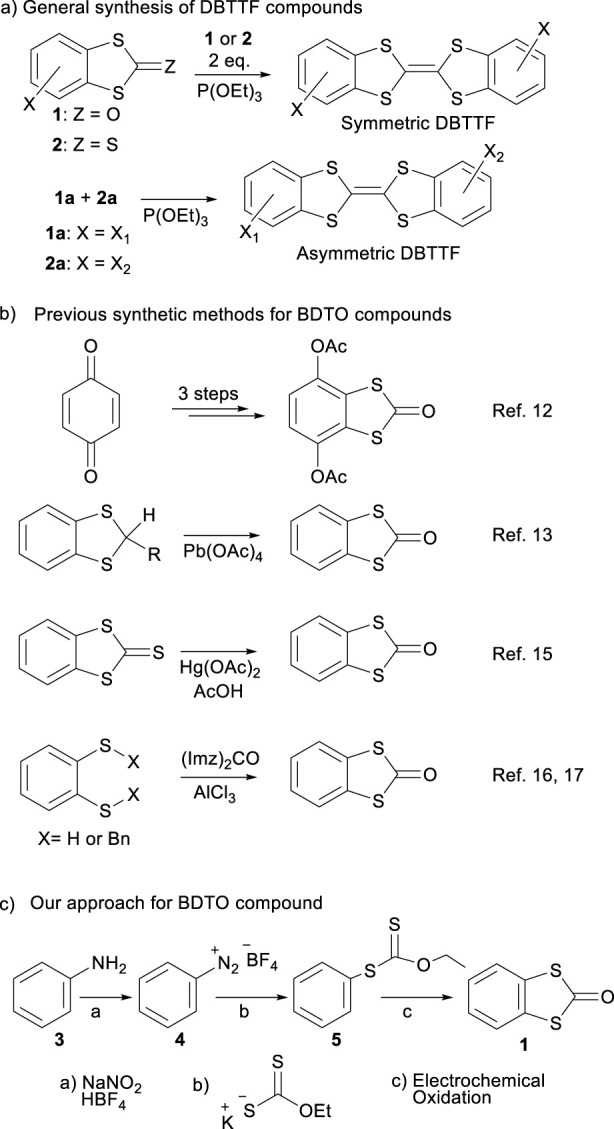
**(a)** Last step in DBTTFs preparation; **(b)** Synthetic routes for obtaining DBTO derivatives; **(c)** New synthetic route developed for the synthesis of DBTO (**1**).

The final coupling from both to yield the symmetrical DBTTF ring is carried out using these intermediates in combination with P(OEt)_3_ as a coupling reagent (Scheme 1a); this final reaction has good yields (80%–90%) (Fanghänel et al., 1995; Inayoshi and Ono, 2000). Besides, one equivalent of BDTO (**1**) and one equivalent of BDTT (**2**) are needed to obtain asymmetric DBTTFs using the same reaction (Gao et al., 2006).

Looking closer at the scarce synthetic routes of BDTO (Scheme 1b), Watson, and Col. (Sun et al., 1997). synthesized **1** derivatives from benzoquinone via 1,4-addition of dithiocarbamate anion to the 6-member ring followed by chemical oxidation. The obtained products contain the p-hydroquinone moiety in the benzene ring, limiting the reaction’s scope. Compound **1** can also be prepared using oxidative decomposition of 1,3-benzodithiol-2-alkyl derivatives (Aromdee et al., 1983) with moderated yields using Pb(OAc)_4_ or DDQ (Nakayama et al., 1977). BDTT has also been used as an intermediate to obtain BDTO via an atom replacement reaction using Hg(OAc)_2_/AcOH under chloroform reflux (de Mayo et al., 1979). The most used reaction to access this important heterocyclic ring requires 1,2-benzeneditiol, Takamasa and Takashi (2002) or 1,2-bis(*S*-benzylthio)-benzene derivatives, Loosli et al. (2005) and carbonylimidazole. All the routes described are long, use corrosive, toxic, and sensitive reagents (Gao et al., 2007) like carbonyl imidazole, prepared from phosgene and readily water reactive, and have a low atom economy (loss of two BnS moieties, for example). Due to the complexity of preparing BDTO derivatives, the most used routes for preparing DBTTFs involve BDTT intermediates (Inayoshi et al., 2016; Senga et al., 1997). Unfortunately, these are not readily prepared either.

Over the last decade, interest in electrosynthesis has increased due to its ability to access unusual functionalities and reactivity, as well as its role in promoting greener and more sustainable synthesis (Cembellín and Batanero, 2021; Frontana-Uribe et al., 2010; Pollok and Waldvogel, 2020). This method also enables the generation of *in situ* reactive intermediates useful in synthetic protocols. Our work focuses on developing a new synthetic route to obtain benzo[d]-1,3-dithiolen-2-one (BDTO) in a simple, fast, and direct manner through electrochemical activation of O-ethyl-*S*-phenyldithiocarbonate, which is derived from the corresponding benzene diazonium salt (Scheme 1c). This article presents our approach and initial findings in an ambitious program aimed at creating a broad range of DBTO products, leveraging the fact that aniline derivatives are widely substituted, affordable, and commercially available precursors for organic synthesis, using green chemistry techniques with significant time and cost savings.

## 2 Materials and methods

### 2.1 General

Commercially purchased reagents were used as starting materials without any additional purification steps. The supporting electrolyte salts were dried in the oven for at least one night before use. Anhydrous-grade solvents were used for cyclic voltammetry and preparative electrolysis experiments. For purification by column chromatography (CC), silica gel (70–230 mesh) and technical grade solvents, but previously distilled, were used. TLC analysis was carried out using Merck TLC Silica gel 80 F254 aluminum sheets.

Melting points were not corrected and carried out on a Fisher-Scientific 12–144 melting point apparatus. NMR spectra were recorded on Bruker (300 MHz) using TMS as an internal reference for ^1^H (0.0 ppm) and CDCl_3_ for ^13^C (77.16 ppm). All the prepared and isolated compounds are known and fit with the reported physical and spectroscopic description. Voltammetric studies were carried out using a PGSTAT204 Potentiostat and a conventional glass cell of 10 mL. Reference electrode: Ag/Ag^+^ (filled with AgNO_3_ 0.01 M in CH_3_CN). Working electrode: glassy carbon disk (diameter: 3 mm). Counter electrode: platinum wire (99.95% purity).

### 2.2 Synthesis of the *O*-ethyl-*S*-phenyldithiocarbonate (5)

*O*-ethyl-*S*-phenyldithiocarbonate (**5**) was prepared in two steps using a variation of the Leuckart methodology, 4; Leuckart and Prakt (1889), Cox et al. (1960) via diazonium tetrafluoroborate salt (**4**). The crystalline solid obtained can be stored in the dark at low temperatures to prevent decomposition. In a typical experiment, 0.5 mL (1 equiv.) of aniline was reacted with tetrafluoroboric acid (2.1 equiv.) added dropwise under constant magnetic stirring, forming a white precipitate corresponding to the anilinium salt. The temperature was maintained at 0 °C and kept under a nitrogen atmosphere. A cold solution (0 °C) of sodium nitrite (1.1 equiv.) in water was slowly added and stirred for 40 min. The precipitate was rapidly recrystallized from an acetone-ether mixture, yielding the product 4 in 83%.

For the second step, several solvents with different polarities were tested as reaction media (Table 1). The reaction cell temperature was set at 45 °C, and 460 mg (2.87 mmol, 1.1 equivalents) of potassium *O*-ethylxanthogenate was dissolved in 6 mL of the respective solvent. In another flask, 100 mg of **4** (0.52 mmol, 1 equivalent) was dissolved in the same volume of solvent. The solution containing compound **4** was added dropwise to the reaction cell and stirred for 1 hour. The reaction produced two major products (**5** and DPDS), which were purified by chromatography (Hexane/AcOEt Mixtures).

**TABLE 1:** Synthesis of *O*-ethyl-*S*-phenyldithiocarbonate **5** in different solvents.

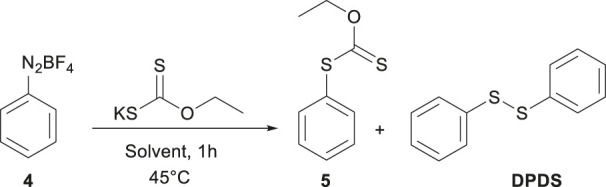			

Entry	Solvent	5 yield (%)	DPDS yield (%)
1	H_2_O	55	4
2	H_2_O/ACN	30	25
3	ACN	20	30
4	DMSO	12	36
5	THF	3	41

### 2.3 General preparative electrolysis of *O*-ethyl-*S*-phenyldithiocarbonate (5), preparation of BDTO

Preparative electrolysis was performed potentiostatically at 1.55 V with 100 mg of compound **5** in an H-type cell at 20 °C. Two glassy carbon plates (4.2 cm^2^) served as the working and counter electrodes, with an Ag/Ag^+^ reference electrode and 0.1 mol L^-1^ NBu_4_PF_6_ in 5 mL of ACN. Each reaction involved degassing both sides with N_2_ for 15 min before initiating the current; the cell was kept under an inert atmosphere. After electrolysis, solvents were removed under vacuum using rotary evaporation, and products were separated by chromatography on a silica gel column (230–400 mesh) with a Hexane–AcOEt mixture. For the other experiments described in Table 2, only the solvent was changed to 25% CH_2_Cl_2_/75% ACN and 25% HFIP/75% ACN.

**TABLE 2:** Electrosynthesis of compound BDTO one in different solvents.

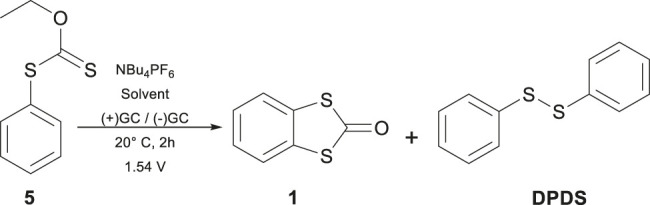				

Entry	Solvent	*E*(V)	BDTO 1 (%)	DPDS (%)
1	ACN	1.55	30	15
2	ACN	1.50	20	25
3	ACN	1.60	22	30
4	ACN/CH_2_Cl_2_	1.55	10	23
5	HFIP/ACN	1.55	35	10
6	HFIP/ACN	1.60	25	27

## 3 Results and discussion

The key intermediate *O*-ethyl-*S*-phenyldithiocarbonate (**5**) was obtained by reacting benzene diazonium tetrafluoroborate salt (**4**) with potassium *O*-ethylxanthogenate; the one-pot reaction was not efficient, and it was necessary to purify **4** to obtain good yields. It was reported that the diazonium tetrafluoroborate salt is more stable than other anions, (Firth and Fairlamb, 2020), and other counter ions were not prepared. The reaction was solvent-dependent, but using H_2_O, the best yield was obtained (Table 1). Interestingly, with more polar solvents, the yield of the target compound increased and the secondary compound DPDS decreased; in low-polarity solvents, this tendency is reversed. A plausible explanation for the presence of DPDS is shown in the (Supplementary Scheme S1). Neutral free radicals are generally considered unaffected by the solvent. However, in cases such as thiyl radicals, the solvent can significantly alter radical reactivity and influence the reaction outcome. Therefore, water promotes the formation of **5** via an ionic mechanism and reduces homolytic fragmentation that triggers radical reactions and the formation of DPDS. Ito and colleagues (Ito and Matsuda, 1982; Ito and Matsuda 1984) showed that thiyl radical intermediates are highly stable, and the recombination rate constant decreases as solvent polarity increases, supporting our observations.

**FIGURE 1:**
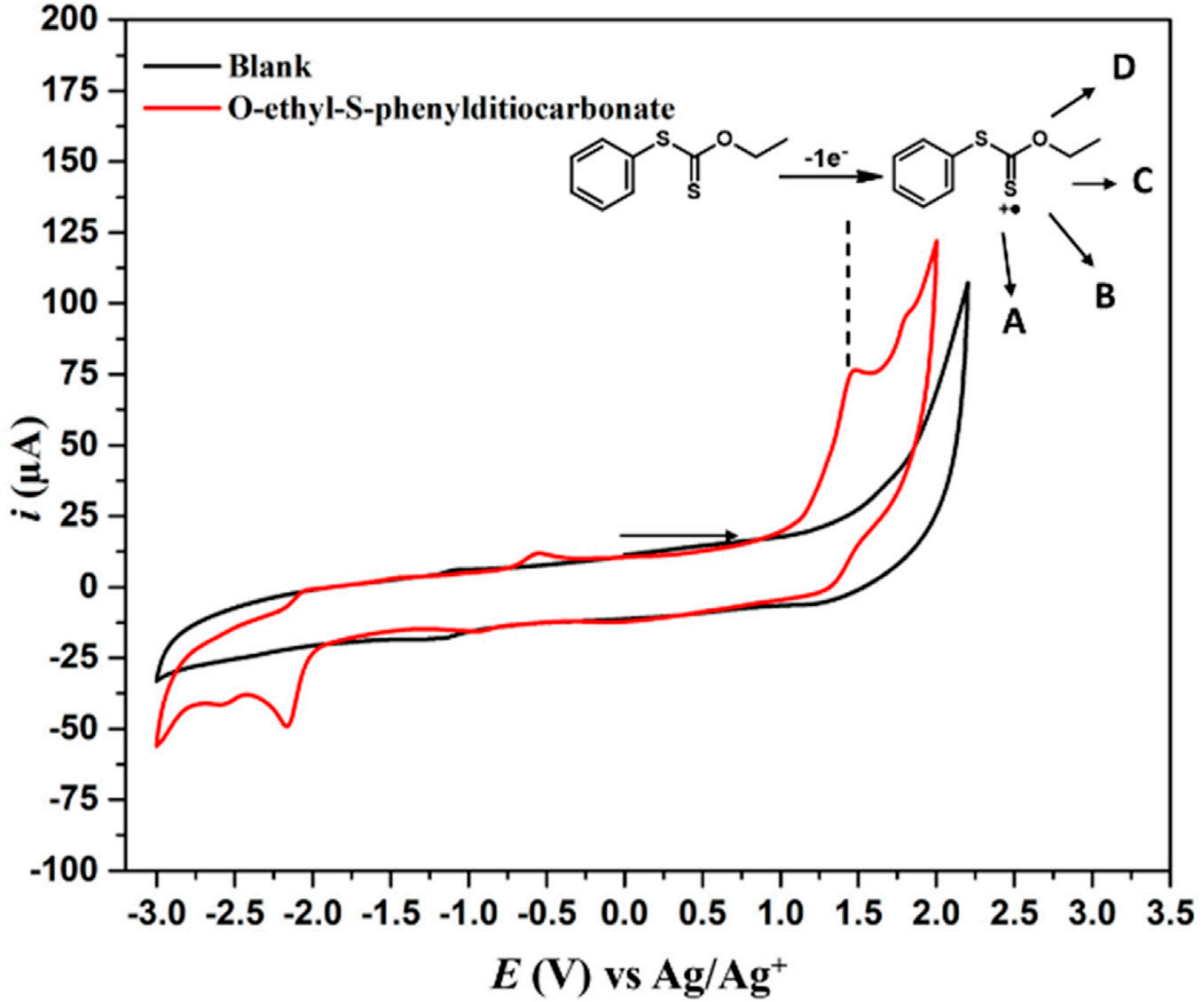
CV of **5** (0.5 mmol L^−1^) in ACN, 0.1 mol L^−1^ NBu_4_PF_6_, *v* = 100 mV s^−1^, WE: Glassy Carbon, RE: Ag/Ag^+^, CE: platinum wire.

**SCHEME 2:**
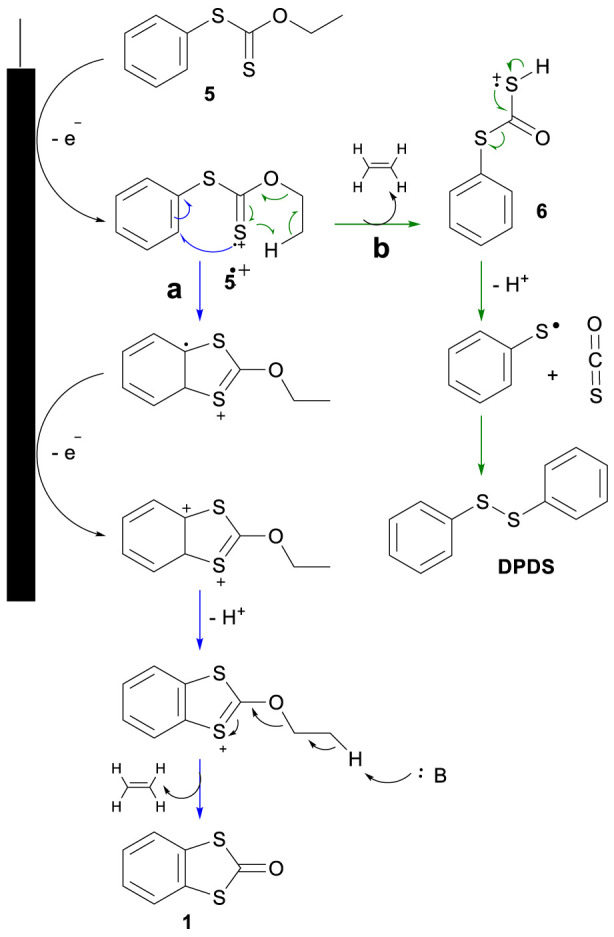
Plausible mechanism for the preparation of BDTO, route a) in blue arrows, and for the preparation of DPDS, route b) in green arrows.

The cyclic voltammetry of *O*-ethyl-*S*-phenyldithiocarbonate (**5**) in the range of −3 V–2 V (Figure 1) showed a well-defined oxidation signal at 1.54 V and 1.84 V, both irreversible. Likewise, two signals are observed at −2.17 V and −2.6 V in the reduction zone, exhibiting irreversible behavior. The anodic signals did not alter with the presence of 1,1,1,3,3,3-hexafluoro-2-propanol (HFIP) as cosolvent (see Supplementary Material); HFIP can stabilize cationic reactive species and produce positive changes in the reactivity (Yoshida et al., 2003). It was hypothesized that the radical cation obtained by the first anodic electron transfer could trigger an intramolecular ring closure to yield the BDTO compound (Scheme 2a). Thus, preparative electrolysis was carried out potentiostatically at 1.55 V (Table 2).

The potentiostatic electrolysis at 1.55 V resulted in multiple products; however, two were found after column separation in a significant proportion, one being the desired product BDTO (**1**) (White crystals from hexane/EtOAc m.p. uncorr. 77 °C-78 °C, lit.15, 78 °C-78.5 °C; ^1^H NMR (see Supplementary Material), agrees with the reported spectra, He et al., 2025; Smith et al., 1989) and a subproduct, the compound DPDS, yielding 30% and 15%, respectively. As in our study, it is proposed that the radical cation intermediate electrochemically produced favors the reaction generally obtained at temperatures greater than 300 °C. The use of HFIP (Ramos-Villaseñor et al., 2020) as cosolvent with ACN (25:75) to generate microdomains that stabilize the cation radical, (Fioroni et al., 2001; Yoshida et al., 2003), just slightly increases the obtained yield to 35%, and the presence of DPDS is still detected. These results imply that these two main reactions compete after forming the radical cation intermediate **5** ^**•+**^ (Scheme 2). Other experimental conditions attempted did not give a better yield of **1**.

To increase the yield, other electrolysis modes could be used, for example, rapid alternating polarity, pulsated alternating electrolysis, which have shown the possibility of avoiding by-products and controlling the reactivity pathway of electrogenerated intermediates (Atkins and Lennox, 2024; Rodrigo et al., 2021). The scalability of this reaction can be envisaged using electrochemical flow reactors with the most promising mode of electrolysis; this technique is well known in industrial processes and has been successfully used in the pharmaceutical industry (Gabriele, 2025).

Scheme 2 depicts the two possible routes to the observed products. On the one hand (Route a), the intramolecular cyclization, which follows a second electron transfer and ethylene extrusion to yield BDTO (**1**), and, on the other (Route b), the elimination of ethylene from intermediate **5** ^**•+**^ and proton transfer to the thiocarbonyl group through a six-member ring intermediate to produce the radical cation **6**. Its decomposition generates carbonyl sulfide and a phenylthiyl radical, which produces the obtained DPDS. This last pathway can be seen as a Chugaev-like elimination reaction, but interestingly favored at room temperature. Classical Chugaev reaction generally occurs at 150 °–200 °C (Tschugaeff and Dtsch, 1900). The fact that this reaction operates at 20 °C can be explained by a process activated by the electron transfer, which produces an activated species similar to the Newman-Kwart reaction, electrochemically favored recently described by Francke et al., (Roesel et al., 2020)^,^ which also occurs at room temperature. Investigations into this new reaction for constructing the benzo[d]-1,3-dithiolen-2-one (BDTO) are ongoing in our lab to increase yield and clarify the scope, and the results will be reported in due time.

## 4 Conclusion

The developed approach introduces a new method to directly obtain the BDTO ring from aniline and serves as a formal synthesis of DBTTF. After three steps, BDTO was synthesized with an overall yield of 16%. Electrochemical analysis showed that phenylxanthate is electroactive, displaying irreversible signals during both oxidation (1.5 V) and reduction (−2.4 V). The electrochemical cyclization of O-ethyl-S-phenyldithiocarbonate (**5**), a key step in this synthesis, produced the BDTO ring with moderate yields of up to 35% (using HFIP/ACN). However, a competing process between electrochemical cyclization and an intramolecular hydrogen transfer, which results in diphenyl disulfide, limited the yield. It is also worth noting that the hazards and drawbacks of using aryldiazonium salts to produce aryl xanthate **5** can now be avoided through an alternative photoactivated route recently published by Wang. (Zhang et al., 2022). The method developed involves a two-step reaction, similar to the diazonium salts method. First, preparation of the aryldibenzothiophenium salt from the corresponding aryl compound using dibenzothiopheneoxide, trifluoroacetic acid, and boron trifluoride etherate. The yields of most compounds reported are good to very good. The second step is the photoactivation of the electron donor-acceptor complex formed by the aryldibenzothiophenium salt and the ethylxanthogenate under 390 nm radiation (purple LED) to produce the aryl xanthate. This method is robust, working with a large variety of functional groups and not requiring the presence of a functional group or protecting groups, as it involves C-H functionalization of the aromatic ring. Following this methodology, the key intermediate, the aryl xanthate **5**, is produced in good yield without the explosion risk associated with the work of large amounts of aryl diazonium salts. Thus, the production of BDTO can be achieved in an orthogonal way, because a large group of functional groups is tolerated during the preparation of the heterocyclic ring. This makes the electrochemical method an attractive way to generate DBTO and, subsequently, the DBTTF system.

## Data Availability

The original contributions presented in the study are included in the article/Supplementary Material, further inquiries can be directed to the corresponding author.
