# Supplementary material for: Formal synthesis of dibenzotetrathiafulvalene (DBTTF), through practical electrochemical preparation of benzo[d]-1,3-dithiole-2-one (BDTO)
Source: Front Chem. 2025 Sep 4;13:1666772. doi: 10.3389/fchem.2025.1666772 (PMC12445048; doi:10.3389/fchem.2025.1666772)
Supplement: Supplementary file 1 [file DataSheet1.docx]

Supplementary Material

**Formal synthesis of dibenzotetrathiafulvalene (DBTTF), through practical electrochemical preparation of benzo[d]-1,3-dithiole-2-one (BDTO)**

**Álvaro V. Terán-Alcocer,^1,2^ Fernanda M. J. Cifuentes-Ajuchan,^3^ Byron J. López-Mayorga^3^ and Bernardo A. Frontana-Uribe^1,2^ * Corresponding Author:** [**bafrontu@unam.mx**](mailto:bafrontu@unam.mx)

# Electrochemical behavior of *O*-ethyl-*S*-phenyldithiocarbonate (5) in different mixtures of HFIP/ACN

The microdomains were expected to form a cluster that could limit the elimination reaction, promoting intramolecular cyclization (Figure S1). HFIP is volatile, slightly corrosive, irritant, and costly; therefore, minimizing its use as a co-solvent is important to reduce risks significantly. Cyclic voltammetries of compound **5** were performed with various HFIP/ACN mixtures to determine the minimum HFIP concentration that favors the reaction. The optimal solvent mixture was found to be 25:75 HFIP/ACN. The complete analysis is shown below.

**Figure S1. Expected control of the competing reactions of phenylxanthate radical cation 5^•+^ and formation of microdomains through the HFIP cosolvent.

The experiment was developed in an electrochemical cell with a three-electrode arrangement, where glassy carbon (3 mm) was used as the working electrode, platinum wire as the counter electrode, and an Ag/Ag^+^ non-aqueous electrode as a reference with a 0.1 mol·L^-1^ NBu_4_PF_6_ supporting electrolyte. Cyclic voltammetries of *O*-ethyl-*S*-phenyldithiocarbonate (**5** 0.5 mmol·L^-1^) were performed in ACN or different HFIP/ACN mixtures to find the minimum concentration of HFIP to carry out the reaction. The mixtures went from 100% to 25% of HFIP at 100 and 500 mV·s-1 (Figure S2).


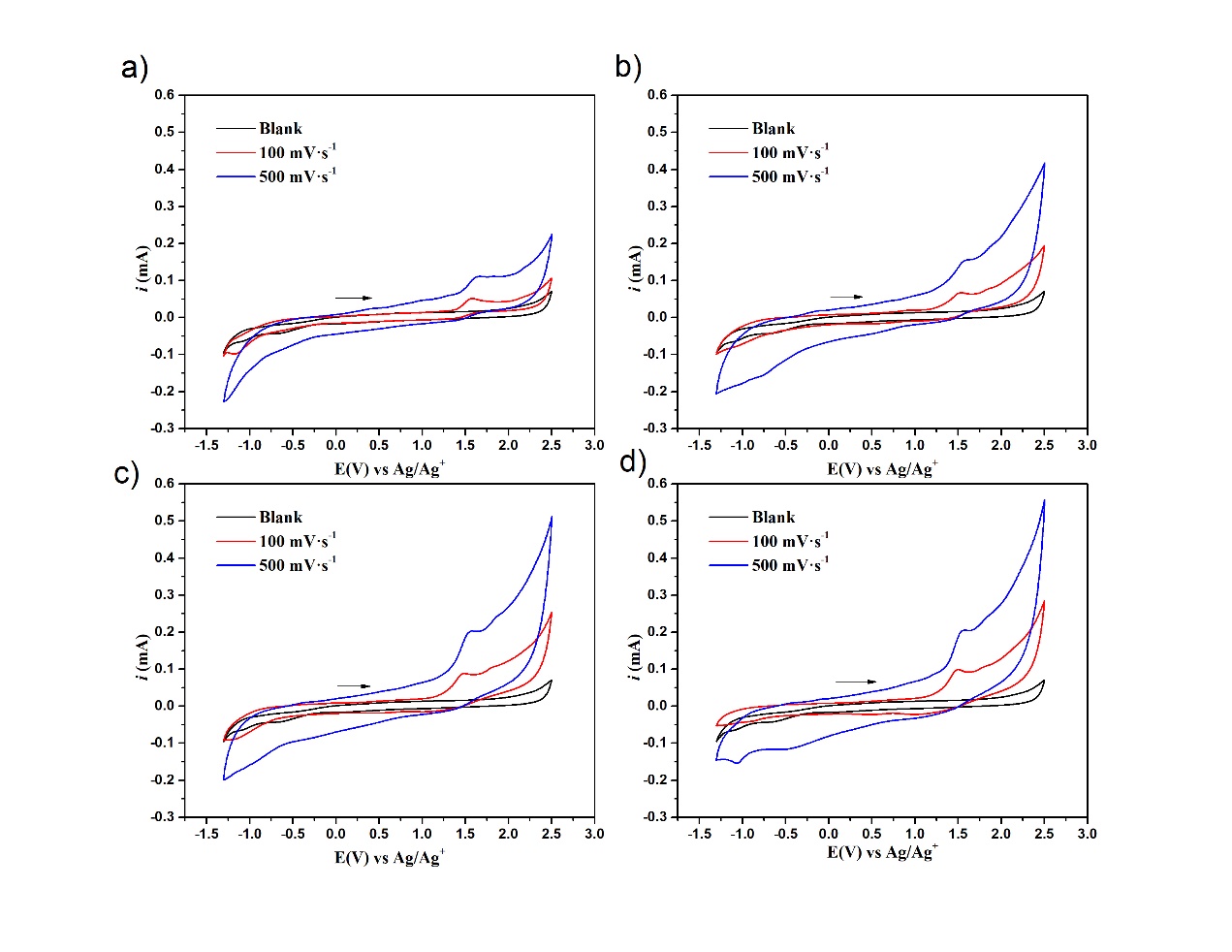


Figure S2. CV´s of compound 5 to evaluate its behavior with different HFIP/ACN mixtures: a) 100:0, b) 75:25, c) 50:50, d) 25:75, v = 100 and 500 mV·s^-1^, 0.1 mol·L^-1^ NBu_4_PF_6_, WE: glassy carbon, RE.: Ag/Ag^+^, CE: platinum, E_i_ = 0 V.

It is observed that the oxidation signal stays at 1.54 V regardless of the HFIP concentration. However, there is a notable change in the peak current. As the HFIP concentration decreases, the oxidation peak current increases (Figure S3).


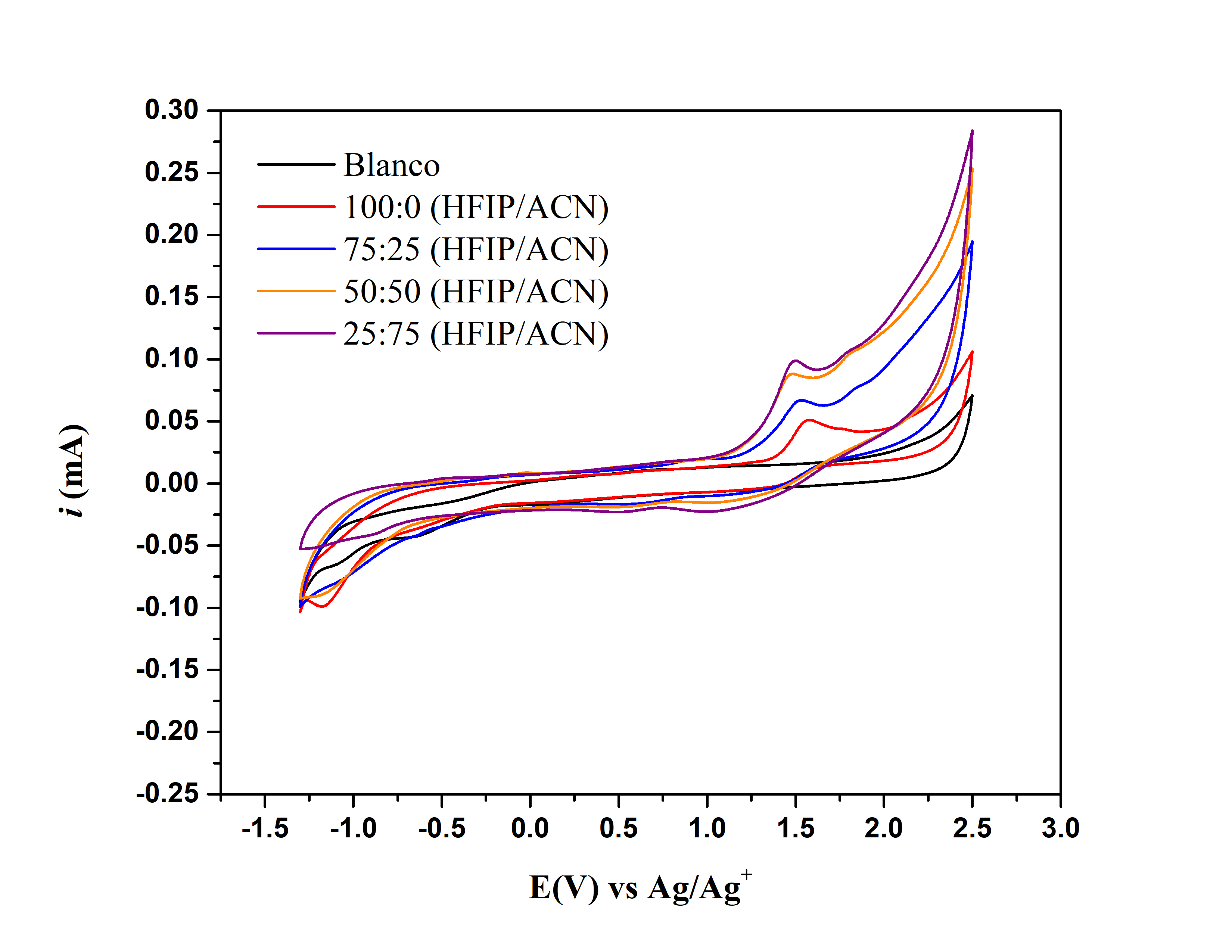


Figure S3. Peak current response of compound 5 (0.5 mmol·L^-1^) with different concentrations of HFIP as cosolvent, v = 100mV·s^-1^, 0.1 mol·L^-1^ NBu_4_PF_6_, WE: glassy carbon, RE: Ag/Ag^+^, CE: platinum, E_i_ = 0 V.

The change in current occurs because of the difference in viscosity of the solvents. HFIP has a viscosity of 1.65 cP at 20°C, while acetonitrile has a viscosity of 0.35 cP at 20°C. Viscosity affects diffusion, which is proportional to the peak current. It is clear that the mixture lowers the viscosity and promotes diffusion. However, the current stops increasing significantly at a 50:50 ratio (HFIP/ACN). Therefore, the 25:75 (HFIP/ACN) mixture, which shows the same response, was selected.

The previously mentioned conditions were used for the electrochemical analysis of compound **5** (0.5 mmol·L-1) in the 25:75 HFIP/ACN solvent mixture. The voltammogram of compound (Figure S4) in the range of -3 V to 2 V shows a well-defined oxidation signal at 1.54 V and another signal at 1.84 V, both irreversible, similar to the response observed in acetonitrile. However, in the reduction zone, the signals at -2.17 V and -2.6 V previously seen in acetonitrile are not visible due to the strong reduction signal of the HFIP solvent, which can produce H₂ through the reduction of the solvent’s acidic protons (pKa = 9.3). Because of this, the electrochemical window narrows on the reduction side, so the optimal working range, to prevent solvent degradation, is approximately from -1.75 V to 2 V.


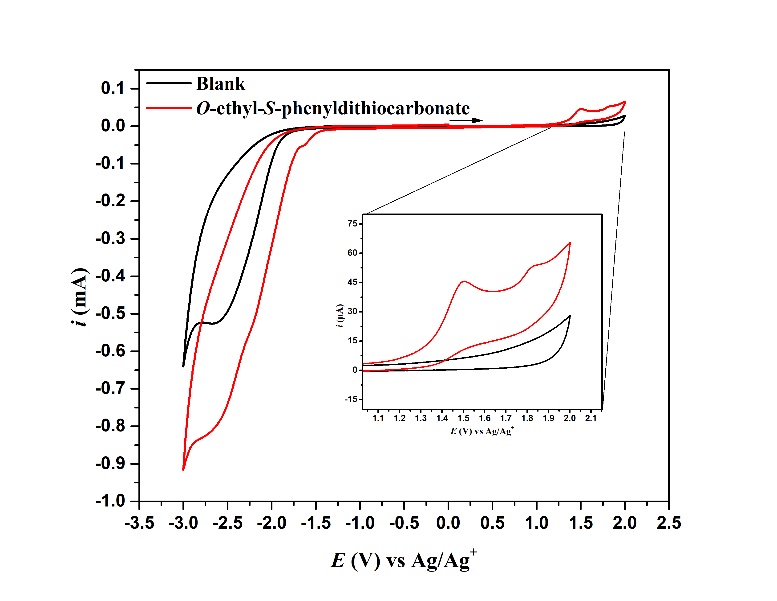


Figure S4. CV of 3 (0.5 mmol·L^-1^) in 25:75 (HFIP/ACN), 0.1 mol·L^-1^ NBu_4_PF_6_, v = 100 mV·s^-1^, WE: glassy carbon, RE: Ag/Ag^+^, CE: platinum, E_i_ = 0 V.

# Mechanistic proposal for the synthesis of *O*-ethyl-*S*-phenyldithiocarbonate (5) and the formation of diphenyl disulfide (DPDS) through the intermediate phenylthiyl radical.

The formation of *O*-ethyl-*S-*phenyldithiocarbonate occurs through either ionic or radical mechanisms, depending on the solvent. The ionic mechanism is favored in polar solvents,^1^ while the radical mechanism is more common in less polar or nonpolar solvents.^2^ Ito and colleagues found that as the polarity of the solvent increases, the stability of the thiyl radical also increases, and the recombination rate constant decreases. Therefore, in water, the formation of *O*-ethyl-*S-*phenyldithiocarbonate via ionic pathways is favored, reducing homolytic fragmentation and inhibiting radical cascade reactions. Conversely, in less polar solvents, the presence of free radicals leads to the formation of phenyl thiyl radicals, which are responsible for creating diphenyl disulfide. These results suggest that the intermediate in the formation of the DPDS compound is a phenylthiyl radical (Scheme SI1).

Scheme SI1. Proposed mechanism for the formation of compound DPDS.

# Spectroscopic information

**Benzenediazonium tetrafluoroborate**


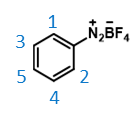


**^1^H-NMR**


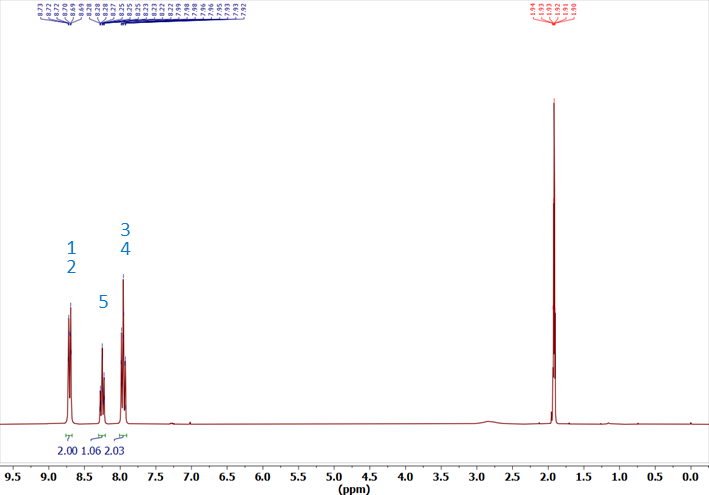


**IR**


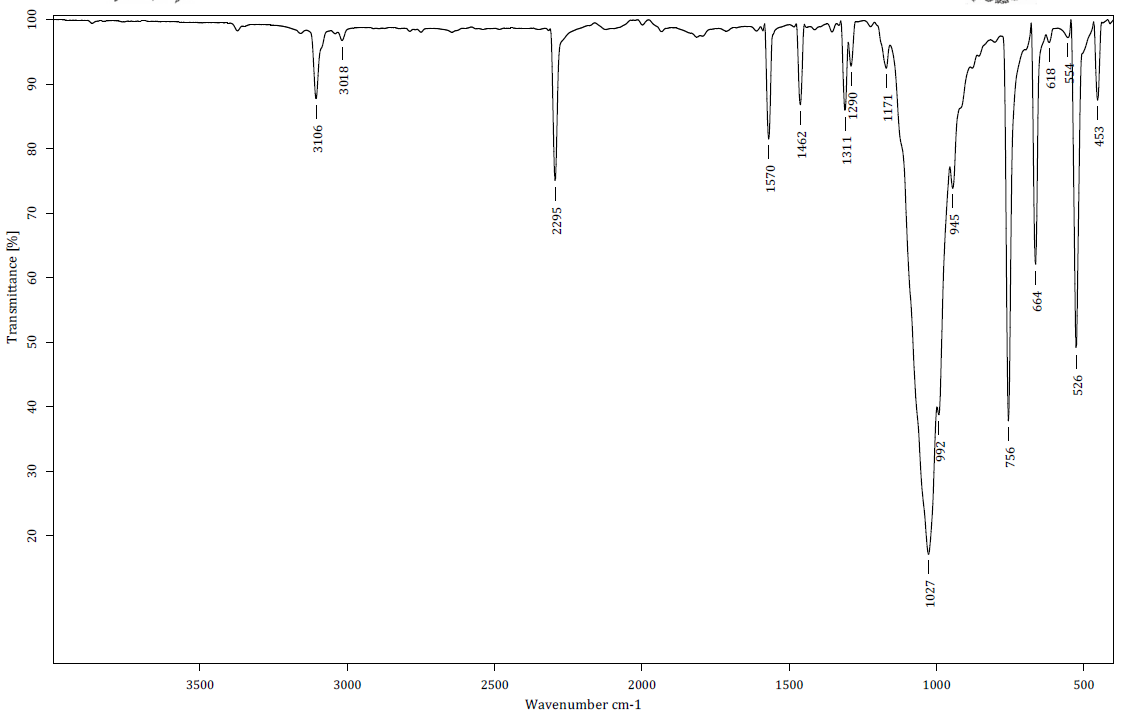


***O*-ethyl-*S*-phenyldithiocarbonate (5)**


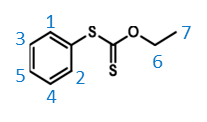


**^1^H-NMR**


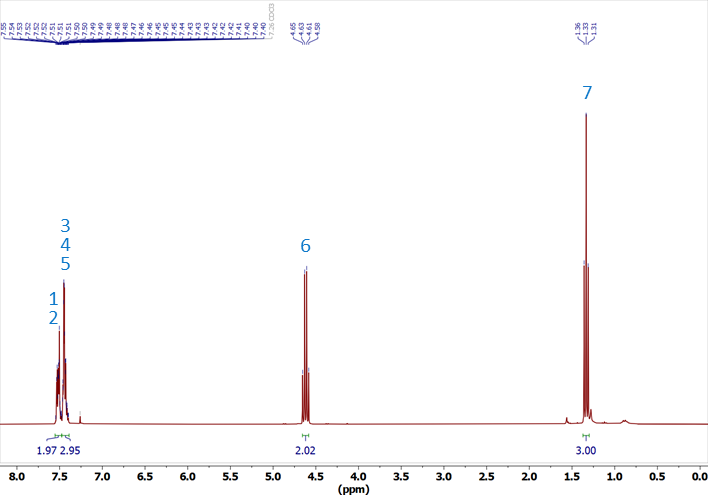


**^13^C-NMR**


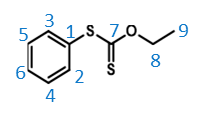


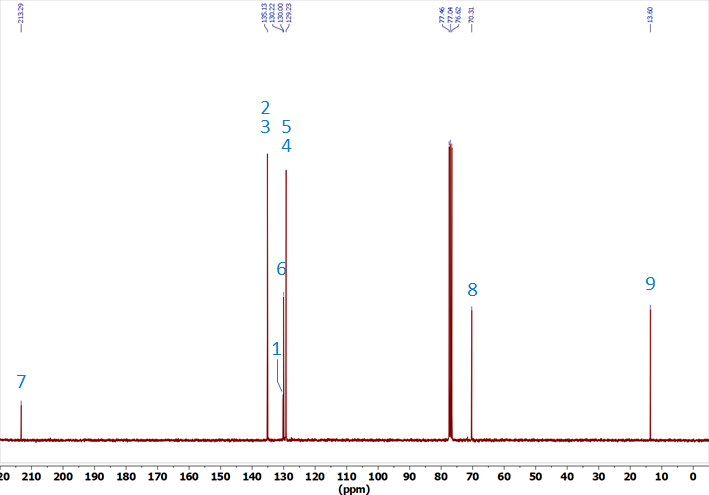


**IR**


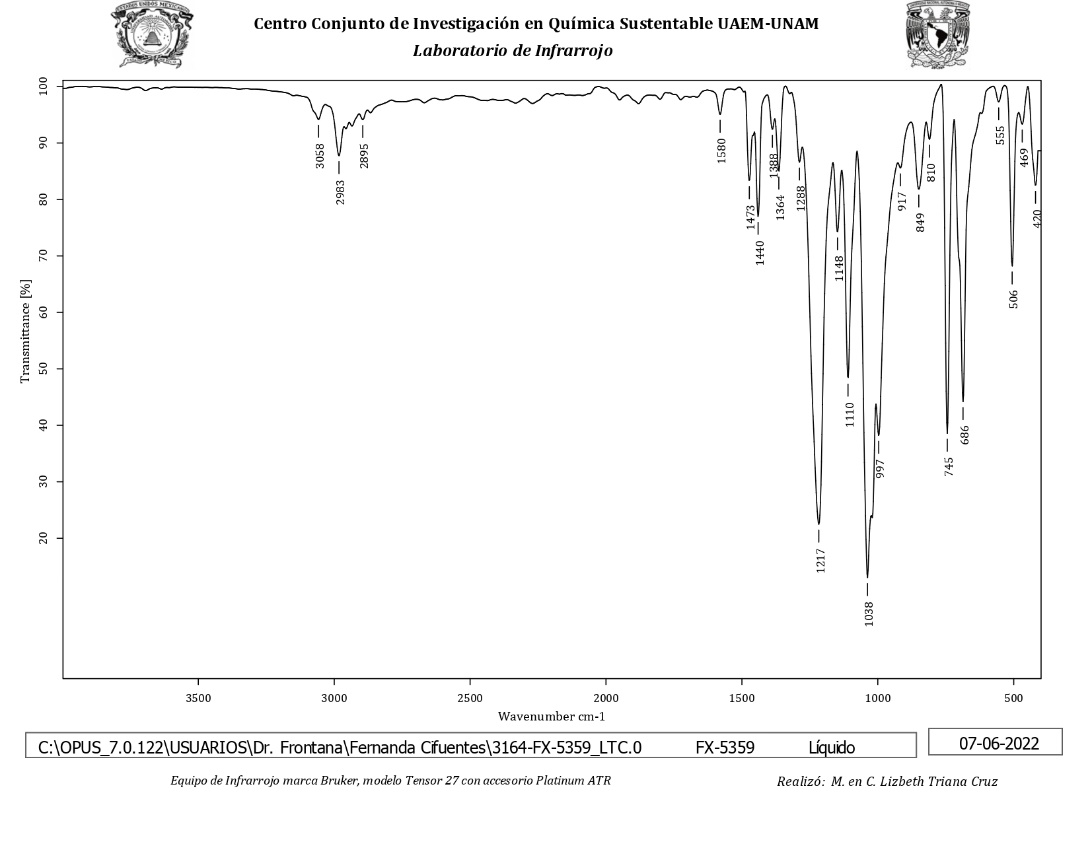


**Diphenyl disulfide (DPDS)**


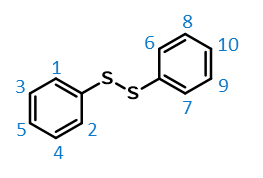


**^1^H-NMR**


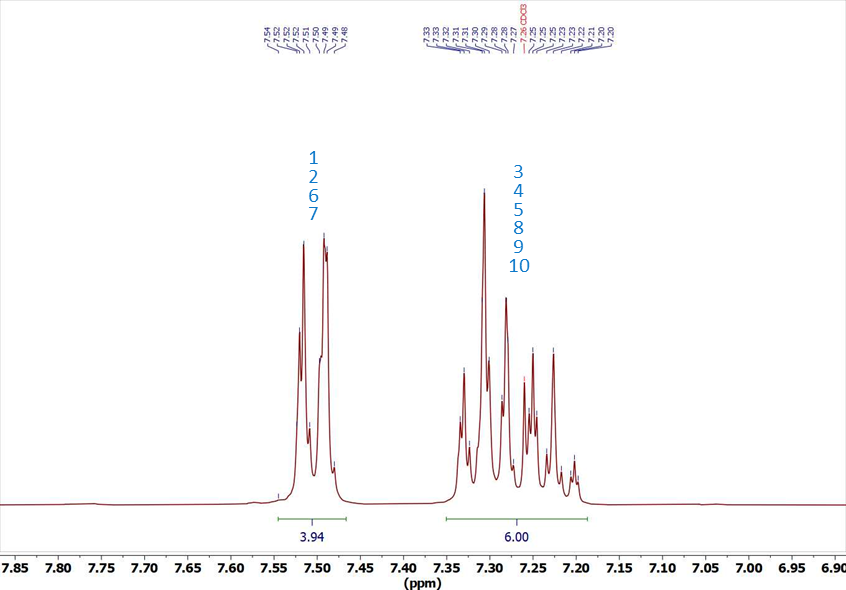


**^13^C-NMR**


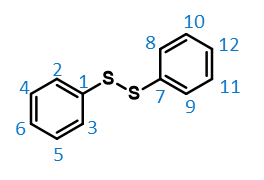


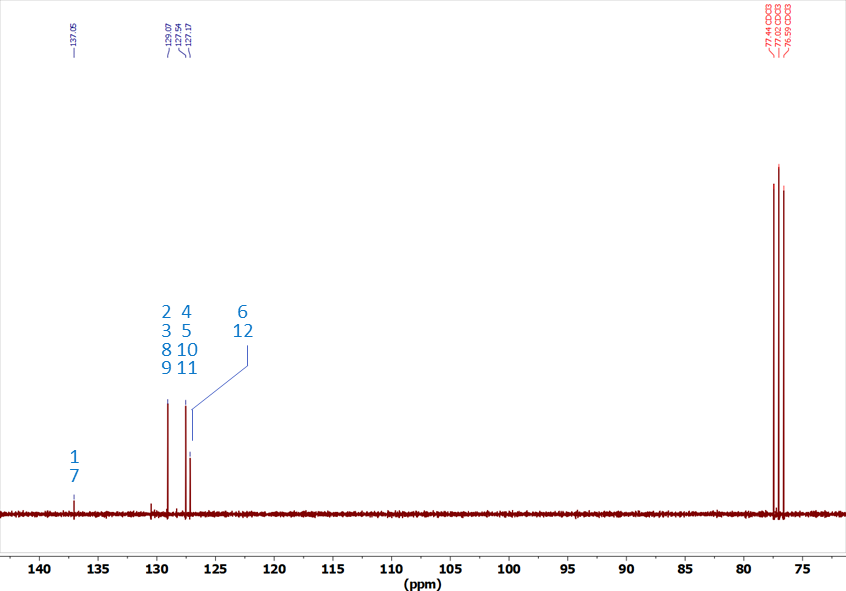


**benzo[d]-1,3-dithiole-2-one (BDTO)^3^**


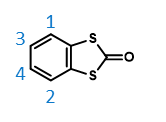


**^1^H-NMR**


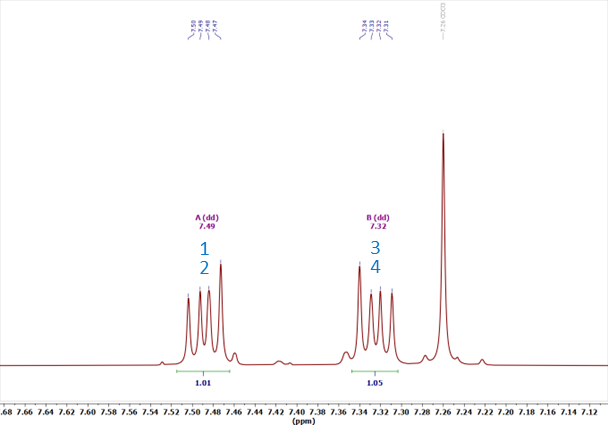


**IR**

**
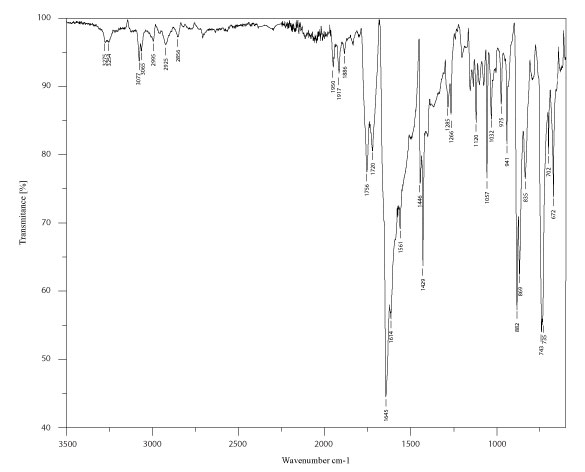
**

# References

1 K. Hölzle, *Helv. Chim. Acta.*, 1946, **29**, 1883

2 L. Tournier, S. Z. Zard, *Tetrahedron Lett.*, 2005, **46**, 971.

3 a) K. Smith, C. M. Lindsay G. J. Pritchard *J. Am. Chem. Soc.* 1989, **111**, 665. b) B-Q. He, L. Zhao, J. Zhang, W-H. Bao, M. Yang, X. Wu. *Angew Chem Int Ed*. 2025; advance online publication. doi:10.1002/anie.202423795
